# Supplementary material for: Structural basis of broad protection against influenza virus by human antibodies targeting the neuraminidase active site via a recurring motif in CDR H3
Source: Nat Commun. 2025 Aug 1;16:7067. doi: 10.1038/s41467-025-62174-2 (PMC12316933; doi:10.1038/s41467-025-62174-2)
Supplement: Supplementary file 1 — Supplementary information [file 41467_2025_62174_MOESM1_ESM.pdf]

Supplementary Information for

Structural basis of broad protection against influenza virus by human antibodies targeting the neuraminidase active site via a recurring motif in CDR H3

Gyunghee Jo[1], Seiya Yamayoshi[2,3,4,5], Krystal M. Ma[6,7,8], Olivia Swanson[1], Jonathan L. Torres[1], James A. Ferguson[1], Monica L. Fernández-Quintero[1], Jiachen Huang[1], Jeffrey Copps[1], Alesandra J. Rodriguez[1], Jon M. Steichen[6,7,8], Yoshihiro Kawaoka[2,4,5,9], Julianna Han[1*], Andrew B. Ward[1*]

[1]Department of Integrative Structural and Computational Biology, The Scripps Research Institute, La Jolla, CA, USA
[2]Division of Virology, Institute of Medical Science, The University of Tokyo, Tokyo, Japan
[3]International Research Center for Infectious Diseases, Institute of Medical Science, University of Tokyo, Tokyo, Japan
[4]Research Center for Global Viral Infections, National Center for Global Health and Medicine, Tokyo, Japan
[5]The University of Tokyo Pandemic Preparedness, Infection and Advanced Research Center (UTOPIA), University of Tokyo, Tokyo, Japan
[6]Department of Immunology and Microbiology, The Scripps Research Institute, La Jolla, CA, USA
[7]IAVI Neutralizing Antibody Center, The Scripps Research Institute, La Jolla, CA, USA
[8]Center for HIV/AIDS Vaccine Immunology and Immunogen Discovery, The Scripps Research Institute, La Jolla, CA, USA
[9]Department of Pathobiological Sciences, School of Veterinary Medicine, University of Wisconsin-Madison, Madison, WI, USA

[*]Correspondence: juliannahan@scripps.edu, andrew@scripps.edu

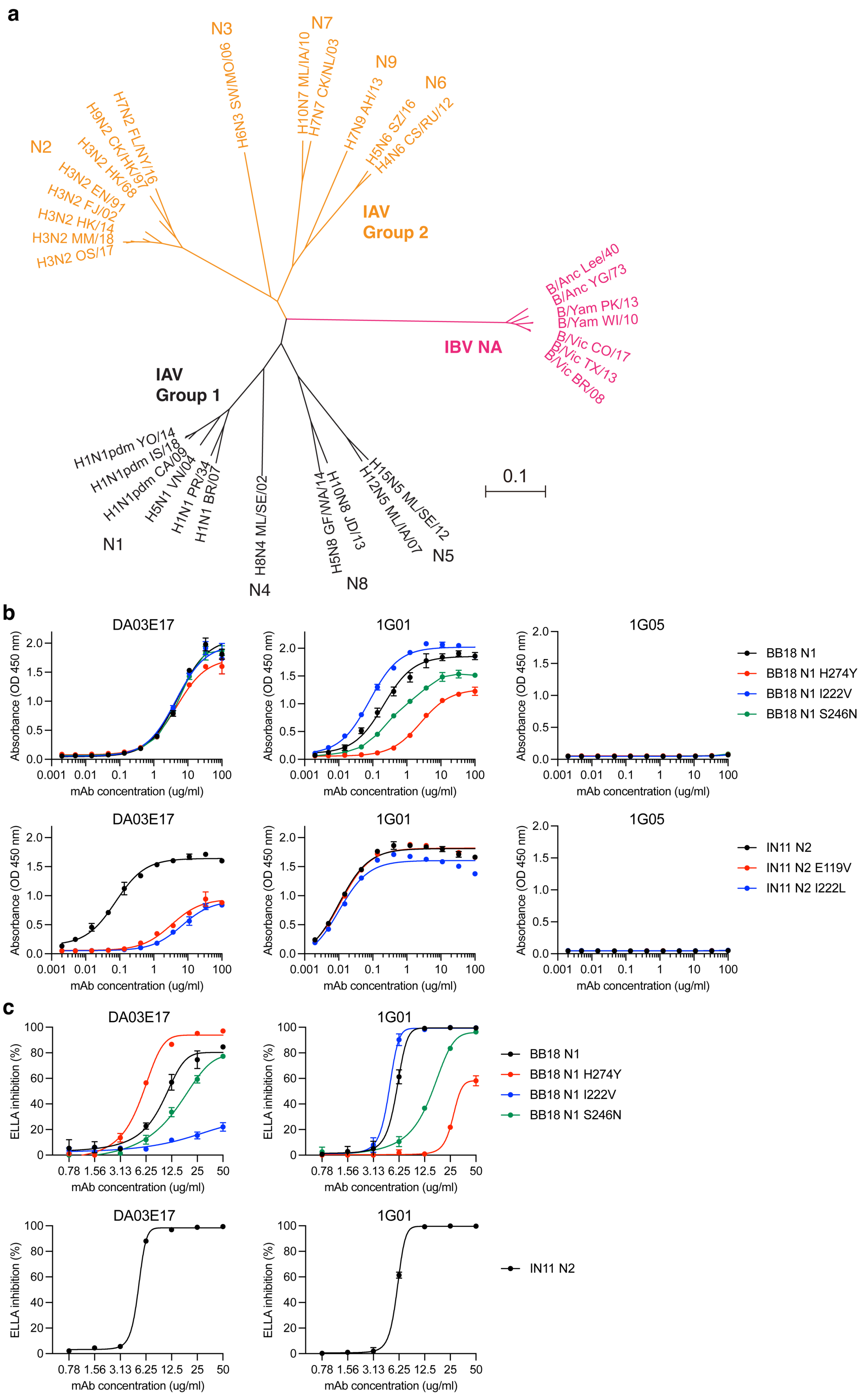

Supplementary Fig. 1. Breadth of mAb DA03E17. a, Phylogenetic tree of influenza A and B virus NAs that DA03E17 showed reactivities (adapted from our previous study[1]). The scale bar represents a 10% change in amino acids. Complete viral strain designations are listed in Source Data file. **b**,**c**, ELISA binding (**b**) and ELLA inhibition (**c**) curves of DA03E17 against recombinant NAs from H1N1 A/Brisbane/02/2018 (BB18 N1 sNAp) and H3N2 A/Indiana/08/2011 (IN11 N2), with or without oseltamivir-resistant mutations. The broadly cross-reactive mAb 1G01 and influenza B virus NA-specific mAb 1G05 were used as positive and negative control, respectively. Data are shown as mean ± SD. Source data are provided as a Source Data file.

a

```
                                          CDR H1
                             26 27 28 29 30 31 32 33
DA03E17 VH:  EVQLVESGPGLVKPSQTLSLTCTVSGGSFSSGGYLWSWVRQHP
IGHV4-31*03: QVQLQESGPGLVKPSQTLSLTCTVSGGSISSGGYYWSWIRQHP
N1 paratope                               ••••
N2 paratope                               ••••
B paratope                                ••••

                        CDR H2
                  51 52 53 54 55 56 57
DA03E17 VH:  GKGLEWIGYILYSGSPYYNPSLESRATISLDTSKNQFSLRLIS
IGHV4-31*03: GKGLEWIGYIYYSGSTYYNPSLKSRVTISVDTSKNQFSLKLSS
N1 paratope            •••••
N2 paratope            •••••
B paratope             ••••

                                   CDR H3
                        93 94 95 96 97 98 99 100 100a 100b 100c 100d 100e 100f 100g 100h 100i 101 102
DA03E17 VH:  VTAADAAMYYCARVDGSGNTDRYYFYGMDVWGQGTMVTVSS
IGHV4-31*03: VTAADTAVYYCAR----------------------------
N1 paratope                 •• ••••••  •
N2 paratope                  • ••••••  •
B paratope                  ••  •••••  •
```

b

```
                                          CDR L1
                            27 28 29 30 31 32
DA03E17 VK:  DIQMTQSPSSVSASVGDRVTITCRASRGIGDWLAWYQQKPGKA
IGKV1-12*01: DIQMTQSPSSVSASVGDRVTITCRASQGISSWLAWYQQKPGKA
N1 paratope                                 •••
N2 paratope                              •• •••
B paratope                               ••   •

                   CDR L2
                 50 51 52
DA03E17 VK:  PKLLIYAASSLQRGVPSRFSGSGSGTDFTLTISSLQPDDFATY
IGKV1-12*01: PKLLIYAASSLQSGVPSRFSGSGSGTDFTLTISSLQPEDFATY
N1 paratope           •
N2 paratope       •                  •
B paratope

                   CDR L3
             89 90 91 92 93 94 95 95a 96 97
DA03E17 VK:  YCQQADGWEVWTFGQGTKVDVK
IGKV1-12*01: YCQQAN----------------
N1 paratope       ••••
N2 paratope       •••
B paratope        ••••
```

c

```
 C  A  R  V  D  G  S  G  N  T  D  R  Y  Y  F  Y  G  M  D  V  W
tgtgcgagagtcgatggttcggggaataccgaccgatattacttctacggcatggacgtctgg
IGHV4-31*03  N1  IGHD3-10*01        N2             IGHJ6*02
```

Total gene-derived nucleotides: 36

Total non-templated nucleotides: 17

Supplementary Fig. 2. Comparison of DA03E17 sequence to its putative germline sequence. a,**b**, Alignment of the DA03E17 variable region sequence with the germline IGHV4-31*03 sequence (**a**), and with the germline IGKV1-12*01 sequence (**b**). The regions that correspond to CDRs are indicated based on the IMGT CDR definition scheme. Residues that differ from the germline are highlighted in red. Residues that interact with each NA are indicated with black dot. **c**, Sequence of VDJ junction of DA03E17 is shown with putative germline gene segments as well as non-templated N-regions indicated. Somatically mutated nucleotides are underlined.

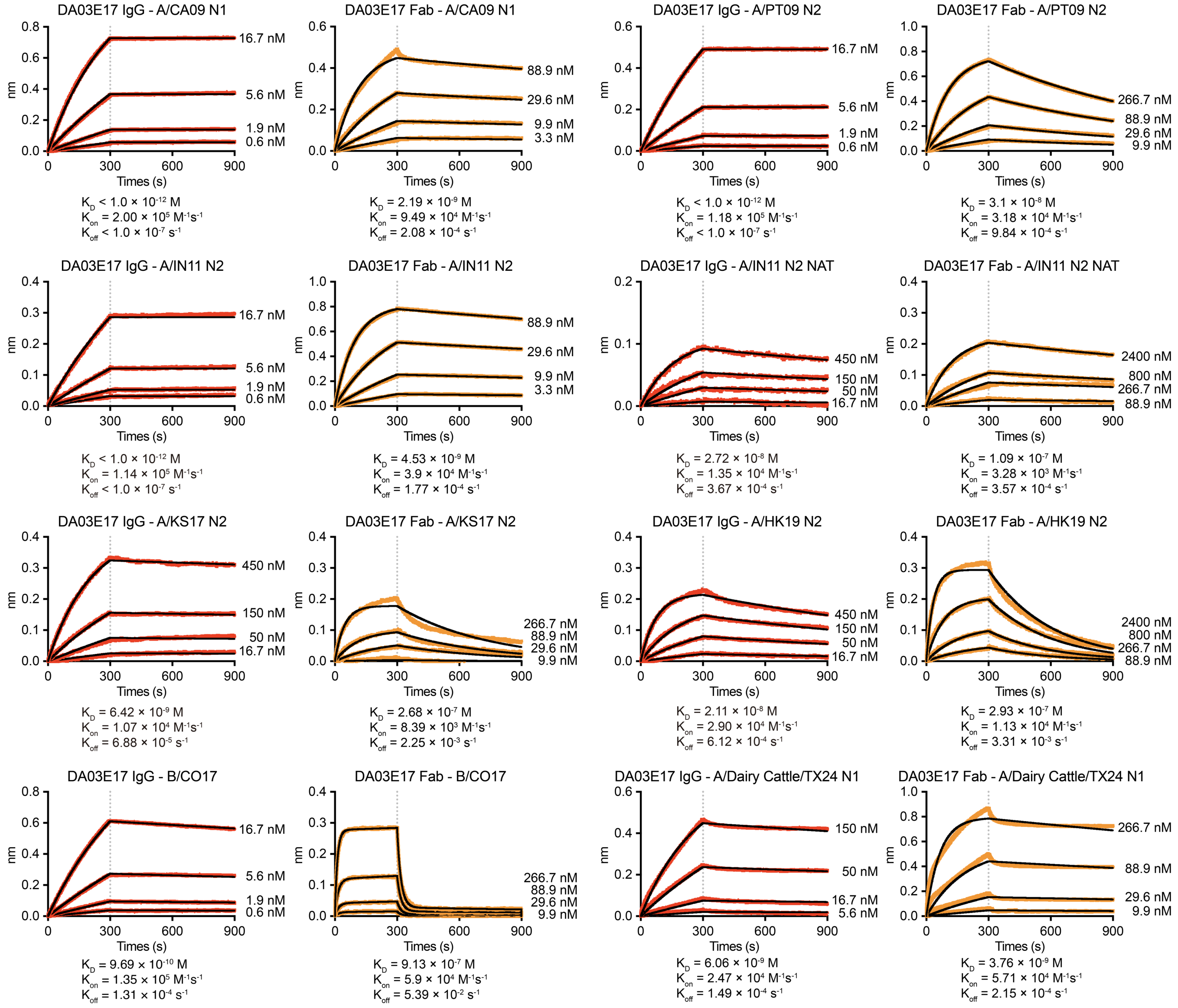

Supplementary Fig. 3. Binding kinetics of DA03E17. Binding of DA03E17 IgG and Fab to recombinant NAs from H1N1 A/California/07/2009 (A/CA09 N1 sNAp), H3N2 A/Perth/16/2009 (A/PT09 N2 NA), H3N2 A/Indiana/08/2011 (A/IN11 N2 NA), H3N2 A/Indiana/08/2011 S245N/S247T mutant (A/IN11 N2 NA NAT mutant), H3N2 A/Kansas/14/2017 (A/KS17 N2 NA), H3N2 A/Hong Kong/2671/2019 (A/HK19 N2 NA), B/Colorado/06/2017 (B/Victoria-lineage; B/CO17 NA), and HPAI H5N1 clade 2.3.4.4b A/Dairy cattle/Texas/24-008749-001/2024 (A/Dairy cattle/TX24 N1 sNAp) as determined by bio-layer interferometry. The experimental data for IgG are shown in red, and for Fab in orange; fitted data are shown in black. A 1:1 binding model was used to estimate K_D values. Source data are provided as a Source Data file.

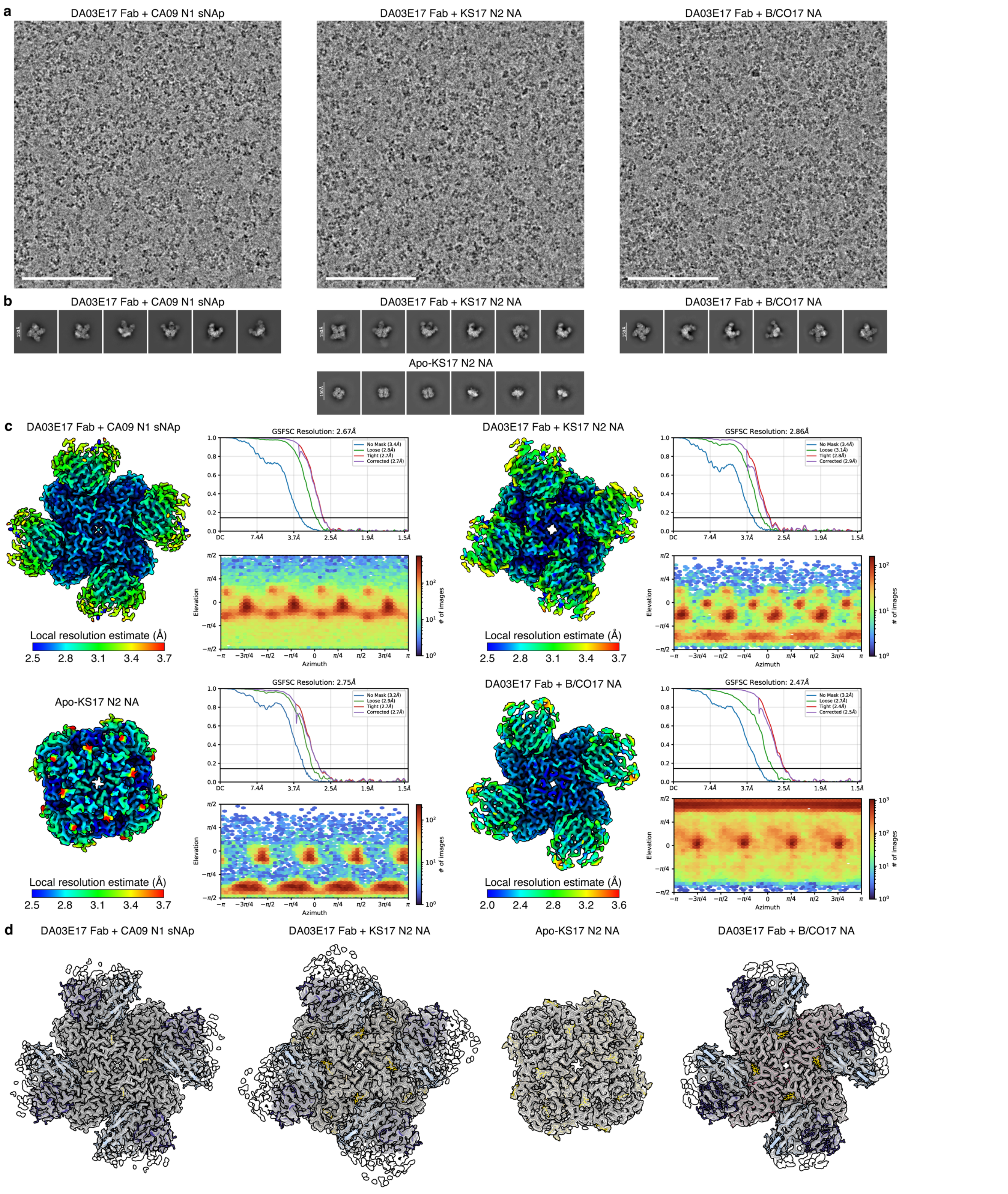

Supplementary Fig. 4. Cryo-EM data processing and validation of DA03E17 in complex with CA09 N1 sNAp, and KS17 N2 and CO17 B NAs. a, Representative micrographs of DA03E17 Fab in complex with CA09 N1 sNAp, and KS17 N2 and CO17 B NAs. Scale bar, 100 nm. **b**, Representative 2D class averages. Scale bar, 150 Å. **c**, Local resolution maps, gold-standard Fourier shell correlation curves, and viewing direction distributions. The 0.143 cutoff is indicated by a horizontal black line. **d**, Map-to-model fits showing final cryo-EM density maps and atomic models.

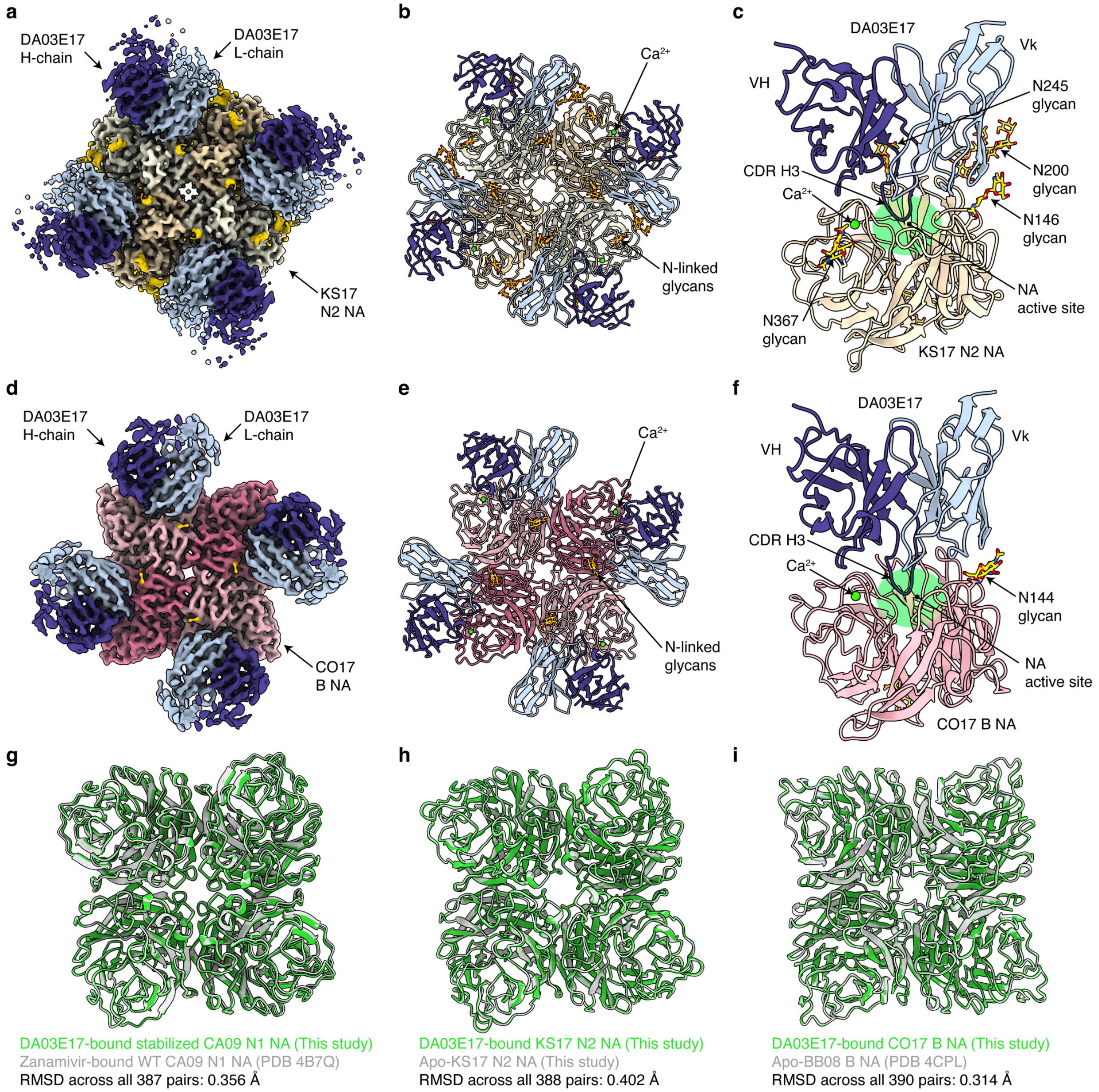

Supplementary Fig. 5. Cryo-EM structures of DA03E17 Fab in complex with KS17 N2 and CO17 B NAs. **a**,**b**, Overall structure of DA03E17 Fab and KS17 N2 NA complex. Cryo-EM map at 2.86 Å (**a**) and atomic model (**b**) from top view. **c**, Ribbon diagram of the KS17 N2 NA protomer bound with one DA03E17 Fab. **d**,**e**, Overall structure of DA03E17 Fab and CO17 B NA complex. Cryo-EM map at 2.47 Å (**d**) and atomic model (**e**) from top view. **f**, Ribbon diagram of the CO17 B NA protomer bound with one DA03E17 Fab. **g**–**i**, Structural comparison between DA03E17-bound N1 sNAp (**g**) and N2 (**h**) and B (**i**) NAs and corresponding WT NAs.

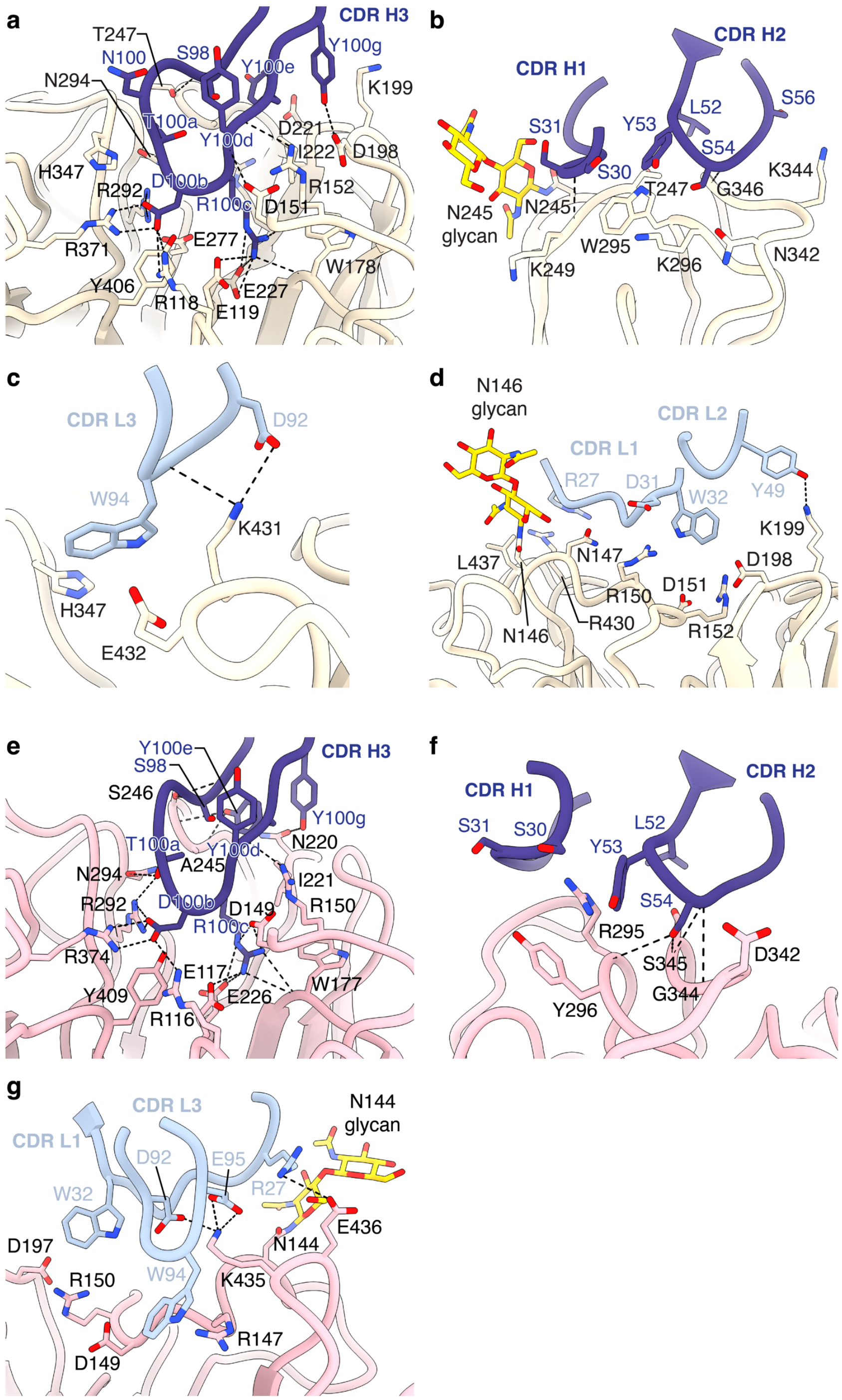

Supplementary Fig. 6. Interactions between DA03E17 and KS17 N2 and CO17 B NAs. a–d, Detailed illustrations of the interactions between KS17 N2 NA and DA03E17 CDR H3 (**a**), CDRs H1 and H2 (**b**), CDR L3 (**c**), and CDRs L1 and L2 (**d**). **e–g**, Detailed illustrations of the interactions between CO17 B NA and DA03E17 CDR H3 (**e**), CDRs H1 and H2 (**f**), and CDRs L1 and L3 (**g**).

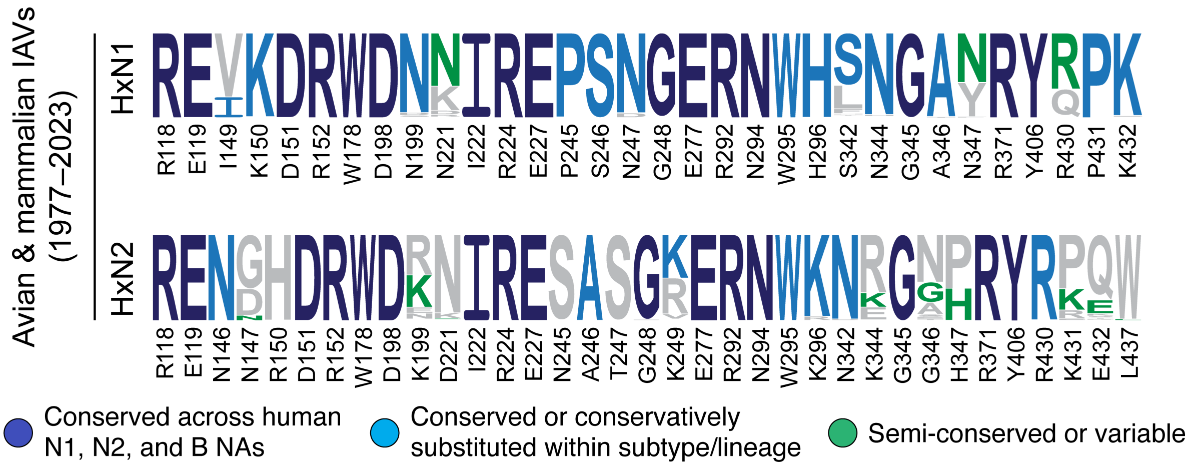

Supplementary Fig. 7. Sequence conservation of the DA03E17 epitope in N1 and N2 NAs from animal-origin IAVs. Epitope residues were analyzed using 432 N1 and 404 N2 NA sequences derived from HxN1 and HxN2 IAVs that have circulated in avian and mammalian hosts between 1977 and 2023. Epitope definitions were based on structural contacts observed in the DA03E17-CA09 N1 and DA03E17-KS17 N2 complexes. Residue coloring follows the scheme used in Fig. 2d: NA residues conserved across human A/H1N1, A/H3N2, and B/Victoria-like viruses are colored in dark blue, while residues conserved or conservatively substituted within each subtype or lineage are shown in sky blue. Semi-conserved or variable residues are colored in green.

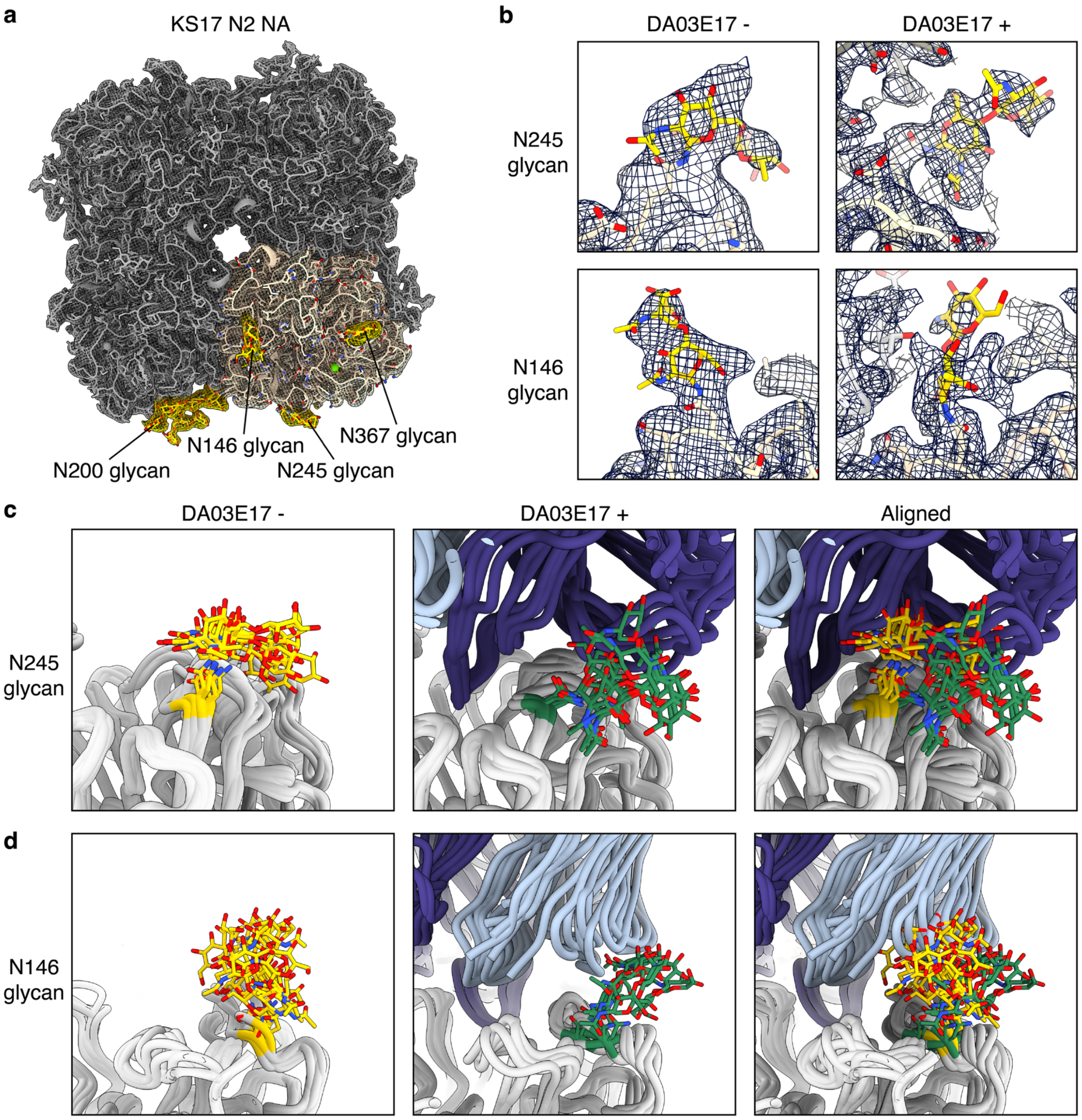

Supplementary Fig. 8. Molecular dynamics simulations of DA03E17-KS17 N2 NA complex. a, Structure of KS17 N2 NA in apo-form with overlaid electron density map. The glycans of one protomer are highlighted in yellow. **b**, Electron density maps (blue mesh) and modeled glycan structures of the N245 and N146 glycans (yellow) in the app-KS17 N2 NA (left) and DA03E17-KS17 N2 NA complex (right) structures, showing conformational changes upon DA03E17 binding. **c**,**d**, Cluster representatives of KS17 N2 NA from MD simulations, shown without DA03E17 bound and with DA03E17 bound, highlighting the structural diversity of the N245 (**c**) and N146 (**d**) glycans in the free state and the population shift of these glycans upon DA03E17 binding.

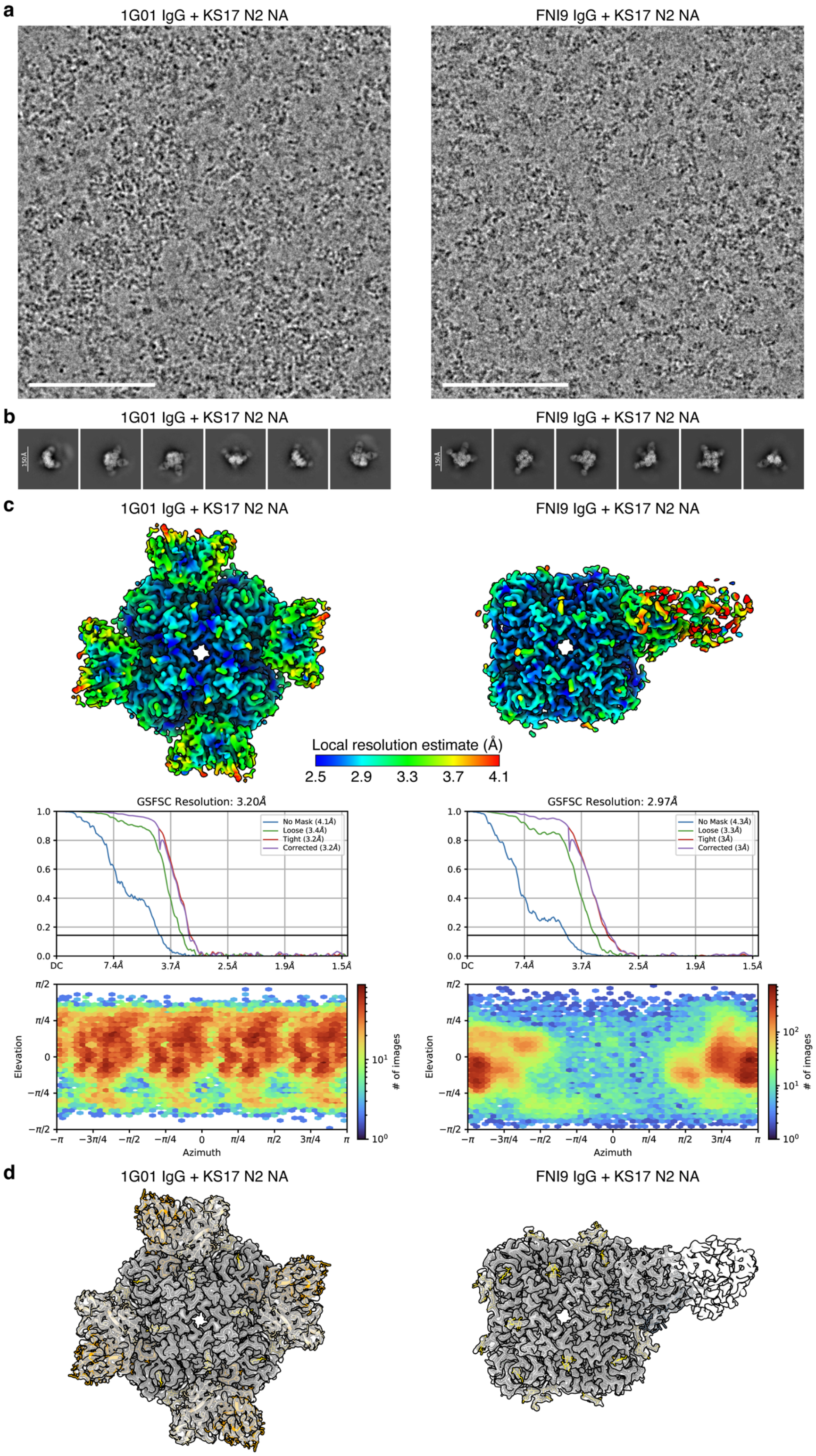

Supplementary Fig. 9. Cryo-EM data processing and validation of KS17 N2 NA in complex with previously reported broadly protective antibodies, 1G01 and FNI9. a, Representative micrographs of KS17 N2 NA in complex with 1G01 IgG and FNI9 IgG. Scale bar, 100 nm. **b**, Representative 2D class averages. Scale bar, 150 Å. **c**, Local resolution maps, gold-standard Fourier shell correlation curves, and viewing direction distributions. The 0.143 cutoff is indicated by a horizontal black line. **d**, Map-to-model fits showing final cryo-EM density maps and atomic models.

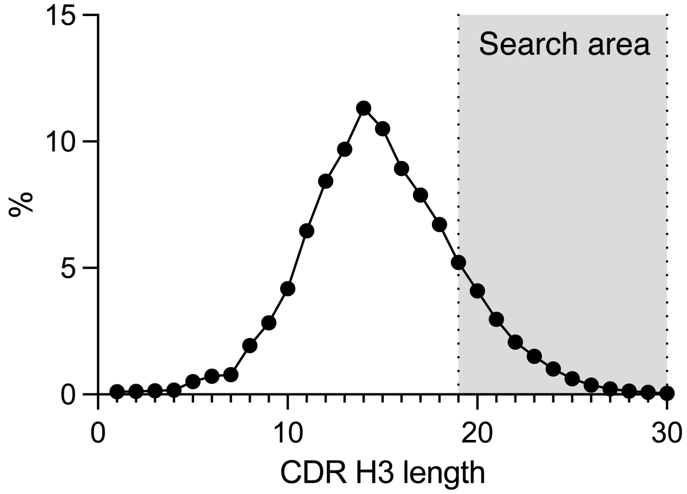

Supplementary Fig. 10. CDR H3 length distribution in the NGS dataset for the 14 human donors. The search area for RD/DR antibodies is shaded gray. Source data are provided as a Source Data file.

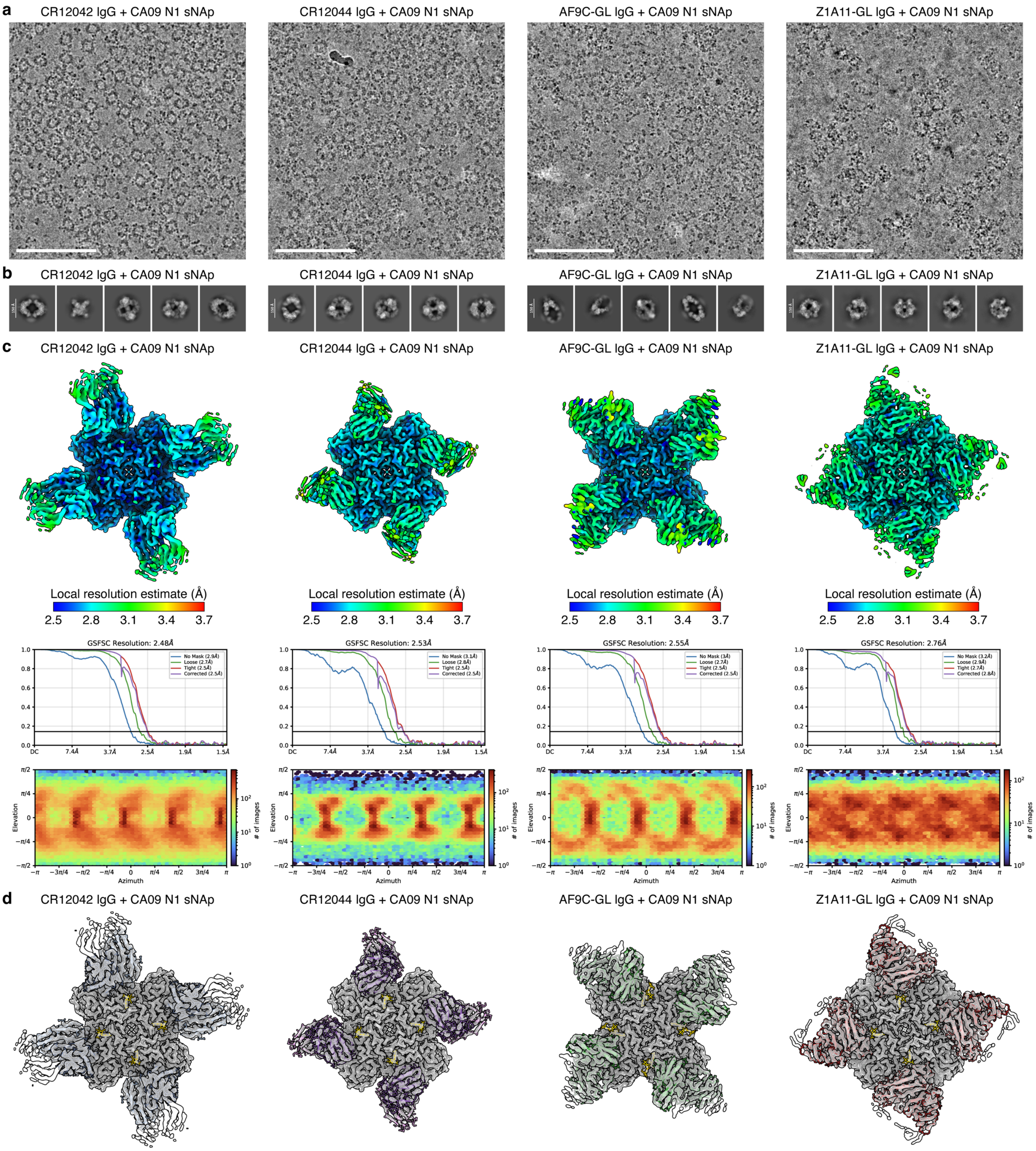

Supplementary Fig. 11. Cryo-EM data processing and validation of additional DR motif antibodies in complex with CA09 N1 sNAp. a, Representative micrographs of additional DR motif antibodies in complex with CA09 N1 sNAp. Scale bar, 100 nm. **b**, Representative 2D class averages. Scale bar, 150 Å. **c**, Local resolution maps, gold-standard Fourier shell correlation curves, and viewing direction distributions. The 0.143 cutoff is indicated by a horizontal black line. **d**, Map-to-model fits showing final cryo-EM density maps and atomic models.

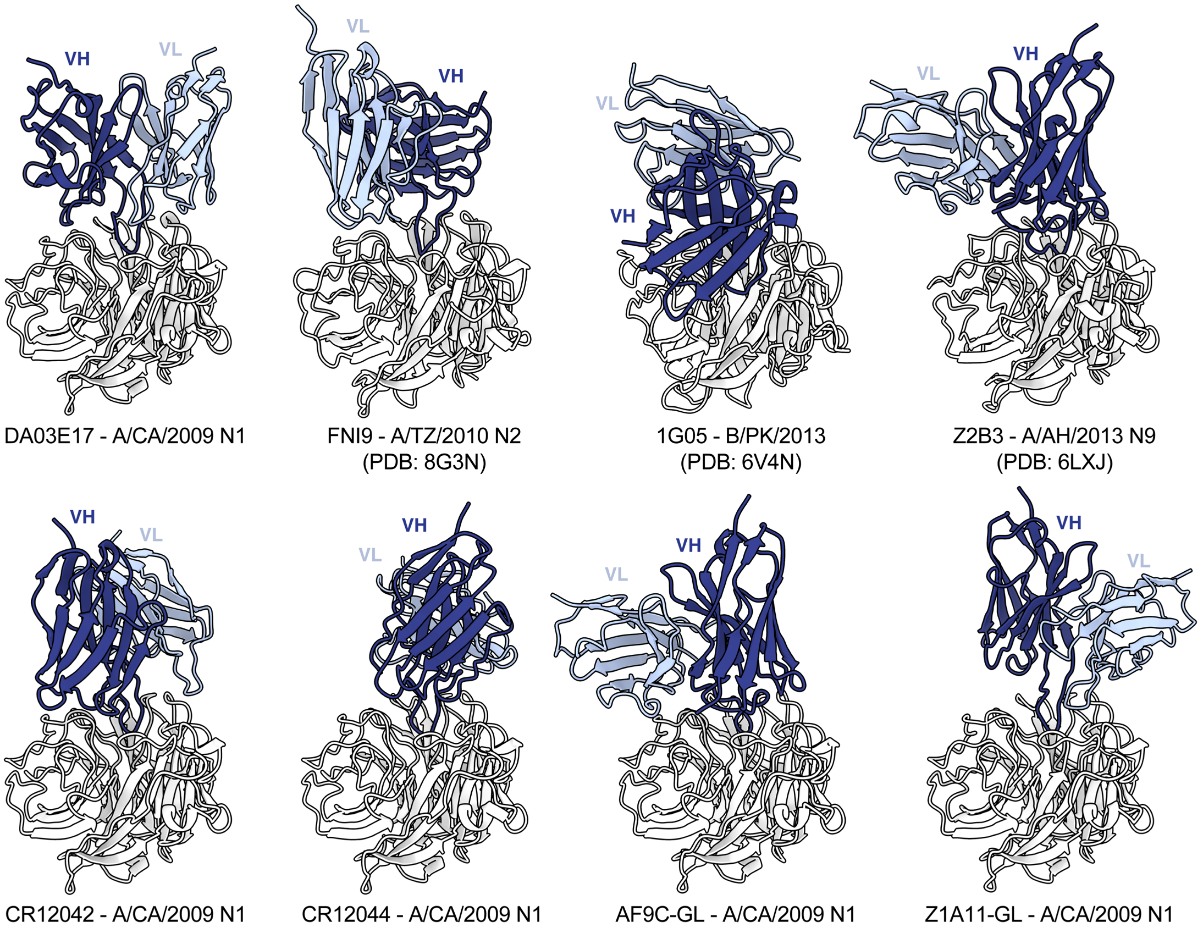

Supplementary Fig. 12. Structural comparison of DR motif NA antibodies. Structures of NA active site antibodies containing a DR motif, identified in this study and in previous studies, are shown aligned by their bound NA protomer and displayed in the same orientation. The variable regions of the heavy and light chains (VH and VL) are colored in dark blue and light blue, respectively.

Supplementary Table 1. Cryo-EM data collection, processing, model refinement and validation statistics.

**Map**	**DA03E17 Fab + CA09 N1 NA**	**DA03E17 Fab + KS17 N2 NA**		**Apo-KS17 N2 NA**		**DA03E17 Fab + CO17 B NA**	**1G01 IgG + KS17 N2 NA**	**FNI9 IgG + KS17 N2 NA**
EMDB	EMD-46041	EMD-46042		EMD-46043		EMD-46044	EMD-46045	EMD-46046
**Data collection**								
Microscope	TFS Glacios	TFS Glacios	TFS Glacios	TFS Glacios	TFS Glacios	TFS Glacios	TFS Glacios	TFS Glacios
Voltage (kV)	200	200	200	200	200	200	200	200
Detector	Falcon 4	Falcon 4	Falcon 4	Falcon 4	Falcon 4	Falcon 4	Falcon 4	Falcon 4
Recording mode	Counting	Counting	Counting	Counting	Counting	Counting	Counting	Counting
Nominal magnification	190,000x	190,000x	190,000x	190,000x	190,000x	190,000x	190,000x	190,000x
Movie micrograph pixel size (Å)	0.725	0.725	0.725	0.725	0.725	0.725	0.725	0.725
Dose rate (e^-/[(camera pixel)*s])	7.68	5.74	5.84	5.74	5.84	5.85	6.54	7.29
EER number of fractions	40	40	40	40	40	40	40	30
Movie micrograph exposure time (s)	3.50	3.99	3.99	3.99	3.99	4.05	3.29	3.18
Total dose (e^-/Å^2)	49.77	42.42	43.16	42.42	43.16	45.06	41.76	45
Defocus range (μm)	-0.7 to -1.4	-0.7 to -1.4	-0.7 to -1.4	-0.7 to -1.4	-0.7 to -1.4	-0.6 to -1.5	-0.7 to -1.4	-0.7 to -1.4
**EM data processing**								
Number of movie micrographs	3,236	5,814		5,894		4,087	3,351	6,228
Number of particle images in map	130,141	47,357		53,710		420,785	51,741	104,786
Symmetry	C4	C4		C4		C4	C4	C1
Map resolution (FSC 0.143; Å)	2.67	2.86		2.75		2.47	3.20	2.97
Map sharpening B-factor (Å^2)	-76.9	-80.4		-87.9		-76.0	-86.1	-64.3
**Structure building and validation**								
Number of atoms in deposited model								
NA	11,920	11,996		11,996		11,064	11,996	11,996
Fab Fv	7,200	7,196		0		7,200	7,408	1,834
Glycans	168	824		808		224	824	822
MolProbity score	1.07	1.05		1.07		1.00	1.19	1.19
Clashscore	1.12	1.13		1.28		0.92	1.29	1.58
Map correlation coefficient, CC (Mask)	0.90	0.87		0.86		0.81	0.78	0.86
EMRinger score	5.71	3.76		3.53		4.45	3.43	4.42
d FSC model (0.5; Å)	2.7	3.0		2.9		2.9	3.5	3.2
RMSD Bonds								
Bond length [Å] (# > 4s)	0.005 (0)	0.006 (0)		0.005 (0)		0.005 (0)	0.006 (0)	0.005 (0)
Bond angles [˚] (# > 4s)	0.863 (8)	0.865 (20)		0.834 (4)		0.910 (0)	0.879 (8)	0.692 (0)
Ramachandran plot								
Favored (%)	96.27	96.47		96.63		96.61	95.16	95.84
Allowed (%)	3.73	3.53		3.37		3.39	4.84	4.16
Outliers (%)	0.00	0.00		0.00		0.00	0.00	0.00
Side chain rotamer outliers (%)	0.76	0.19		0.89		0.77	0.56	0.78
Cβ outliers (%)	0.00	0.00		0.00		0.00	0.00	0.00
PDB	9CYE	9CYF		9CYG		9CYH	9CYI	9CYJ

Supplementary Table 1 (cont'd). Cryo-EM data collection, processing, model refinement and validation statistics.

**Map**	**CR12042 IgG + CA09 N1 NA**	**CR12044 IgG + CA09 N1 NA**	**AF9C-GL IgG + CA09 N1 NA**	**Z1A11-GL IgG + CA09 N1 NA**
EMDB	EMD-70108	EMD-70109	EMD-70110	EMD-70111
**Data collection**				
Microscope	TFS Glacios	TFS Glacios	TFS Glacios	TFS Glacios
Voltage (kV)	200	200	200	200
Detector	Falcon 4	Falcon 4	Falcon 4	Falcon 4
Recording mode	Counting	Counting	Counting	Counting
Nominal magnification	190,000x	190,000x	190,000x	190,000x
Movie micrograph pixel size (Å)	0.725	0.725	0.725	0.725
Dose rate (e^-/[(camera pixel)*s])	7.81	7.74	7.81	7.74
EER number of fractions	40	40	40	40
Movie micrograph exposure time (s)	3.03	3.06	3.03	3.06
Total dose (e^-/Å^2)	45	45	45	45
Defocus range (μm)	-0.7 to -1.7	-0.7 to -1.4	-0.7 to -1.4	-0.7 to -1.4
**EM data processing**				
Number of movie micrographs	2,178	1,684	3,142	2,119
Number of particle images in map	255,311	99,260	107,490	134,231
Symmetry	C4	C4	C4	C4
Map resolution (FSC 0.143; Å)	2.48	2.53	2.55	2.76
Map sharpening B-factor (Å^2)	-78.3	-65.7	-76.8	-91.9
**Structure building and validation**				
Number of atoms in deposited model				
NA	11,920	11,920	11,920	11,920
Fab Fv	7,260	7,008	7,140	7,372
Glycans	376	264	508	264
MolProbity score	0.75	0.76	0.83	0.66
Clashscore	0.79	0.86	1.15	0.45
Map correlation coefficient, CC (Mask)	0.89	0.86	0.87	0.87
EMRinger score	4.76	3.77	3.80	4.05
d FSC model (0.5; Å)	2.6	2.7	2.8	2.9
RMSD Bonds				
Bond length [Å] (# > 4s)	0.005 (0)	0.005 (0)	0.005 (0)	0.005 (0)
Bond angles [˚] (# > 4s)	0.853 (8)	0.777 (0)	0.830 (8)	0.840 (0)
Ramachandran plot				
Favored (%)	98.38	98.38	99.19	99.52
Allowed (%)	1.62	1.62	0.81	0.48
Outliers (%)	0.00	0.00	0.00	0.00
Side chain rotamer outliers (%)	0.38	0.57	0.19	0.93
Cβ outliers (%)	0.00	0.00	0.00	0.00
PDB	9O4N	9O4O	9O4P	9O4Q

Supplementary Table 2. DA03E17 epitope conservation across IAV subtypes and IBV lineages.

Met=1 numbering			DA03E17 CA09 N1 epitope	DA03E17 KS17 N2 epitope	DA03E17 CO17 B epitope	IAV Group 1				IAV Group 2					IBV	
N1	N2	B				N1 (98,463)[a]	N4 (484)	N5 (752)	N8 (6,667)	N2 (135,160)	N3 (2,187)	N6 (5,727)	N7 (1,558)	N9 (3,447)	Victoria lineage (30,156)	Yamagata lineage (14,098)
118	118	116	R	R	R	R[b] (99.99)[c]	R (99.59)	R (99.87)	R (99.96)	R (99.99)	R (100)	R (99.98)	R (100)	R (99.97)	R (99.99)	R (99.99)
119	119	117	E	E	E	E (99.67)	E (100)	E (99.87)	E (99.97)	E (99.92)	E (99.95)	E (99.74)	E (100)	E (99.59)	E (99.98)	E (99.96)
146	146	144	-	N	N	N (99.80)	N (100)	N (100)	S (99.60)	N (95.34)	N (99.68)	N (99.97)	N (100)	N (99.97)	N (99.92)	N (99.93)
147	147	145	-	N	-	G (99.81)	G (100)	N (99.87)	N (99.93)	N (82.72)	G (99.91)	G (99.32)	G (99.42)	G (99.71)	G (99.88)	G (99.91)
149	149	147	I	-	R	I (77.68)	V (99.59)	V (99.34)	V (96.24)	V (82.57)	I (85.56)	I (96.68)	I (97.43)	I (98.99)	R (99.95)	R (99.94)
150	150	148	K	R	-	K (99.70)	K (99.79)	K (99.87)	K (99.39)	R (77.34)	K (98.58)	H (97.59)	H (99.42)	H (99.62)	G (98.93)	E (96.71)
151	151	149	D	D	D	D (99.01)	D (100)	D (100)	D (99.97)	D (95.78)	D (99.31)	D (99.97)	D (100)	D (99.94)	D (99.64)	D (99.77)
152	152	150	R	R	R	R (99.96)	R (100)	R (100)	R (99.91)	R (99.98)	R (99.95)	R (99.83)	R (99.94)	R (99.86)	R (100)	R (99.98)
179	178	177	W	W	W	W (99.99)	W (100)	W (100)	W (99.93)	W (99.88)	W (100)	W (99.98)	W (100)	W (100)	W (100)	W (99.99)
199	198	197	D	D	D	D (99.74)	D (100)	D (100)	D (99.96)	D (99.91)	D (100)	N (96.26)	N (100)	N (99.59)	D (99.82)	D (99.51)
200	199	198	N	K	-	S (53.88)	S (51.34)	D (96.54)	S (56.81)	K (89.04)	N (97.58)	N (96.89)	D (93.77)	N (99.97)	N (99.40)	S (95.39)
222	221	220	N	D	N	N (67.75)	N (98.76)	Q (99.47)	D (99.31)	D (71.66)	D (86.69)	N (99.56)	N (99.61)	N (99.19)	N (93.03)	N (97.43)
223	222	221	I	I	I	I (99.68)	I (98.56)	I (100)	I (99.81)	I (99.65)	I (98.95)	I (99.91)	I (99.94)	I (99.71)	I (99.92)	I (99.82)
225	224	223	R	R	R	R (99.98)	R (100)	R (99.87)	R (99.97)	R (99.97)	R (99.82)	R (99.97)	R (100)	R (100)	R (99.99)	R (99.94)
228	227	226	E	E	E	E (99.97)	E (100)	E (99.87)	E (99.99)	E (99.98)	E (100)	E (99.97)	E (99.94)	E (100)	E (99.99)	E (99.99)
246	245	244	P	N	S	P (99.77)	P (100)	P (100)	P (99.96)	N (65.54)	P (99.95)	P (98.67)	S (98.59)	S (62.98)	S (99.72)	P (95.21)
247	246	245	S	A	A	S (99.52)	S (99.79)	A (99.87)	A (81.97)	A (99.88)	A (99.95)	A (99.83)	A (100)	A (99.74)	A (99.93)	A (99.93)
248	247	246	N	T	S	D (74.86)	D (98.14)	N (89.64)	N (99.57)	T (66.24)	A (86.47)	N (97.96)	S (97.11)	T (99.83)	S (99.83)	S (98.25)
249	248	247	G	G	G	G (97.46)	A (99.79)	N (72.91)	R (98.64)	G (99.55)	N (86.15)	N (89.70)	S (97.88)	G (100)	G (99.63)	G (99.87)
250	249	248	-	K	-	Q (96.02)	Q (100)	Q (99.87)	Q (99.91)	K (91.12)	S (84.92)	R (57.48)	Q (99.74)	P (100)	V (98.74)	I (88.11)
277	276	275	-	-	E	E (99.99)	E (100)	E (99.87)	E (99.99)	E (99.92)	E (100)	E (99.98)	E (100)	E (100)	E (99.97)	E (99.92)
278	277	276	E	E	E	E (99.99)	E (100)	E (99.87)	E (99.99)	E (99.99)	E (100)	E (99.91)	E (100)	E (100)	E (99.98)	E (99.91)
293	292	292	R	R	R	R (99.98)	R (100)	R (100)	R (99.96)	R (99.96)	R (99.95)	R (99.97)	R (100)	R (98.17)	R (99.98)	R (99.95)
295	294	294	N	N	N	N (99.93)	N (100)	N (99.73)	N (99.97)	N (99.98)	N (99.95)	N (99.93)	N (99.94)	N (99.97)	N (99.98)	N (99.94)
296	295	295	W	W	R	W (99.99)	W (100)	W (100)	W (100)	W (99.98)	W (100)	W (99.97)	W (100)	W (100)	R (92.77)	S (85.10)
297	296	296	H	K	Y	H (99.98)	R (97.32)	N (99.60)	T (80.26)	K (99.00)	K (99.73)	K (97.44)	Q (99.94)	Q (99.36)	Y (99.96)	Y (99.88)
339	342	341	S	N	D	S (85.99)	N (98.56)	G (100)	G (99.99)	N (99.98)	S (99.41)	T (92.10)	T (99.42)	P (99.97)	D (96.15)	D (94.54)
341	344	343	N	K	K	N (95.20)	K (99.79)	T (100)	Deletion	K (48.55)	N (86.71)	S (85.81)	P (99.94)	N (97.65)	K (59.91)	E (89.02)
342	345	344	G	G	G	G (99.99)	E (100)	N (98.54)	Q (89.79)	G (99.98)	G (99.95)	P (97.64)	G (99.36)	N (97.80)	G (99.97)	G (99.95)
343	346	345	A	G	S	A (99.54)	R (99.79)	N (99.87)	G (99.30)	G (71.59)	G (85.39)	D (98.12)	A (97.11)	N (99.97)	S (98.78)	S (98.94)
344	347	346	N	H	-	N (85.31)	Y (100)	Y (99.73)	Y (99.90)	H (92.20)	P (85.30)	P (99.88)	P (99.74)	N (98.98)	G (98.74)	G (99.41)
368	371	374	R	R	R	R (99.98)	R (100)	R (99.87)	R (99.66)	R (99.99)	R (100)	R (99.97)	R (100)	R (99.94)	R (100)	R (99.98)
402	406	409	Y	Y	Y	Y (99.98)	Y (100)	Y (99.87)	Y (99.63)	Y (99.99)	Y (100)	Y (99.97)	Y (100)	Y (99.97)	Y (100)	Y (99.99)
430	430	433	R	R	-	R (91.58)	Q (100)	K (99.47)	K (52.17)	R (99.49)	R (84.11)	R (98.62)	R (99.36)	R (92.95)	G (99.80)	G (99.89)
431	431	434	P	K	-	P (99.89)	P (99.79)	P (99.47)	P (100)	K (90.95)	P (99.68)	P (99.84)	P (100)	P (99.97)	G (99.93)	G (99.95)
432	432	435	K	E	K	E (61.35)	K (99.79)	E (98.94)	E (99.85)	E (87.68)	N (99.77)	K (96.28)	E (100)	K (99.51)	K (99.79)	K (99.14)
433	433	436	-	-	E	E (99.90)	E (100)	E (100)	E (99.97)	E (99.92)	K (89.22)	E (99.83)	E (100)	E (99.94)	E (99.49)	T (95.42)
436	437	437	-	L	-	I (97.77)	I (99.79)	I (100)	I (99.60)	L (83.37)	S (99.27)	L (97.84)	W (98.78)	W (99.80)	T (99.93)	T (99.83)

DA03E17 epitope residues conserved across influenza A and B viruses are highlighted in cyan. Epitope residues highly conserved (>90%) within the corresponding IAV group or IBV and within the corresponding subtype or lineage are highlighted in green and yellow, respectively.

[a]Number of influenza NA sequences for each subtype or lineage obtained from the GISAID database (https://www.gisaid.org/) in November 2023.

[b]Most common residue at each position.

[c]Percent identity for most common residue.

Reference

1. Yasuhara, A. et al. A broadly protective human monoclonal antibody targeting the sialidase activity of influenza A and B virus neuraminidases. *Nat Commun* **13**, 6602 (2022).
