# Supplementary material for: Structural basis of broad protection against influenza virus by human antibodies targeting the neuraminidase active site via a recurring motif in CDR H3
Source: Nat Commun. 2025 Aug 1;16:7067. doi: 10.1038/s41467-025-62174-2 (PMC12316933; doi:10.1038/s41467-025-62174-2)
Supplement: Supplementary file 2 — Reporting summary [file 41467_2025_62174_MOESM2_ESM.pdf]

nature portfolio

Corresponding author(s): Andrew B. Ward

Last updated by author(s): Jun 27, 2025

Reporting Summary

Nature Portfolio wishes to improve the reproducibility of the work that we publish. This form provides structure for consistency and transparency in reporting. For further information on Nature Portfolio policies, see our Editorial Policies and the Editorial Policy Checklist.

Statistics

For all statistical analyses, confirm that the following items are present in the figure legend, table legend, main text, or Methods section.

n/a	Confirmed	
☐	☒	The exact sample size (*n*) for each experimental group/condition, given as a discrete number and unit of measurement
☐	☒	A statement on whether measurements were taken from distinct samples or whether the same sample was measured repeatedly
☒	☐	The statistical test(s) used AND whether they are one- or two-sided<br>*Only common tests should be described solely by name; describe more complex techniques in the Methods section.*
☒	☐	A description of all covariates tested
☒	☐	A description of any assumptions or corrections, such as tests of normality and adjustment for multiple comparisons
☐	☒	A full description of the statistical parameters including central tendency (e.g. means) or other basic estimates (e.g. regression coefficient) AND variation (e.g. standard deviation) or associated estimates of uncertainty (e.g. confidence intervals)
☒	☐	For null hypothesis testing, the test statistic (e.g. *F*, *t*, *r*) with confidence intervals, effect sizes, degrees of freedom and *P* value noted<br>*Give P values as exact values whenever suitable.*
☒	☐	For Bayesian analysis, information on the choice of priors and Markov chain Monte Carlo settings
☒	☐	For hierarchical and complex designs, identification of the appropriate level for tests and full reporting of outcomes
☒	☐	Estimates of effect sizes (e.g. Cohen's *d*, Pearson's *r*), indicating how they were calculated

Our web collection on statistics for biologists contains articles on many of the points above.

Software and code

Policy information about availability of computer code

Data collection	EPU software (Thermo Fisher) was used for automated collection of cryo-EM data.
Data analysis	Micrographs were aligned using Patch Motion Correction and CTF estimation was done by Patch CTF in CryoSPARC Live. Micrographs were curated based on CTF fits, with those worse than 6–8 Å excluded due to poor quality. All following processing was done using CryoSPARC v4.2.1 and v4.4.1. For cryo- EM structure modelling, UCSF ChimeraX v1.6, Coot v0.9.8.7, and Phenix v1.20.1 were used. Structural figures were prepared using UCSF ChimeraX v1.6.<br>PySpark scripts used in precursor frequency analysis are available at https://github.com/SchiefLab/Jo2025, along with instructions on setting up an EMR cluster.

For manuscripts utilizing custom algorithms or software that are central to the research but not yet described in published literature, software must be made available to editors and reviewers. We strongly encourage code deposition in a community repository (e.g. GitHub). See the Nature Portfolio guidelines for submitting code & software for further information.

Data

Policy information about availability of data

All manuscripts must include a data availability statement. This statement should provide the following information, where applicable:

- Accession codes, unique identifiers, or web links for publicly available datasets
- A description of any restrictions on data availability
- For clinical datasets or third party data, please ensure that the statement adheres to our policy

The atomic models and cryo-EM density maps generated in this study have been deposited to the Protein Data Bank (PDB) and Electron Microscopy Databank (EMDB) respectively. The accession codes are 9CYE and EMD-46041 (DA03E17 Fab+CA09 N1 NA), 9CYF and EMD-46042 (DA03E17 Fab+KS17 N2 NA), 9CYG and EMD-46043 (Apo-KS17 N2 NA), 9CYH and EMD-46044 (DA03E17 Fab+CO17 B NA), 9CYI and EMD-46045 (1G01 IgG+KS17 N2 NA), 9CYJ and EMD-46046 (FNI9 IgG +KS17 N2 NA), 9O4N and EMD-70108 (CR12042 IgG+CA09 N1 NA), 9O4O and EMD-70109 (CR12044 IgG+CA09 N1 NA), 9O4P and EMD-70110 (AF9C-GL IgG+CA09 N1 NA), and 9O4Q and EMD-70111 (Z1A11-GL IgG+CA09 N1 NA). Source data are provided with this paper.

Research involving human participants, their data, or biological material

Policy information about studies with human participants or human data. See also policy information about sex, gender (identity/presentation), and sexual orientation and race, ethnicity and racism.

Reporting on sex and gender	n/a
Reporting on race, ethnicity, or other socially relevant groupings	n/a
Population characteristics	n/a
Recruitment	n/a
Ethics oversight	n/a

Note that full information on the approval of the study protocol must also be provided in the manuscript.

Field-specific reporting

Please select the one below that is the best fit for your research. If you are not sure, read the appropriate sections before making your selection.

☒ Life sciences ☐ Behavioural & social sciences ☐ Ecological, evolutionary & environmental sciences

For a reference copy of the document with all sections, see nature.com/documents/nr-reporting-summary-flat.pdf

Life sciences study design

All studies must disclose on these points even when the disclosure is negative.

Sample size	Sample size is not applicable as this study did not include in vivo experiments.
Data exclusions	No data were excluded from the analysis.
Replication	The ELISA and ELLA experiments were replicated two times. The cryo-EM, BLI, and MD experiments were not explicitly replicated.
Randomization	Randomization is not applicable as this study did not include in vivo experiments.
Blinding	Blinding is not applicable as this study did not include in vivo experiments.

Reporting for specific materials, systems and methods

We require information from authors about some types of materials, experimental systems and methods used in many studies. Here, indicate whether each material, system or method listed is relevant to your study. If you are not sure if a list item applies to your research, read the appropriate section before selecting a response.

Materials & experimental systems

n/a	Involved in the study	
☐	☒	Antibodies
☐	☒	Eukaryotic cell lines
☒	☐	Palaeontology and archaeology
☒	☐	Animals and other organisms
☒	☐	Clinical data
☒	☐	Dual use research of concern
☒	☐	Plants

Methods

n/a	Involved in the study	
☒	☐	ChIP-seq
☒	☐	Flow cytometry
☒	☐	MRI-based neuroimaging

Antibodies

Antibodies used	HRP-conjugated goat anti-human IgG Fc secondary antibody (Southern Biotech, Cat. No.: 2048-05)
Validation	Secondary antibodies were bought from commercial vendors and were validated by the manufacturers. (https://www.southernbiotech.com/goat-anti-human-igg-fc-hrp-2048-05)

Eukaryotic cell lines

Policy information about cell lines and Sex and Gender in Research

Cell line source(s)	Expi293F cells (Thermo Fisher Scientific)
Authentication	The cell lines were not authenticated since they were purchased commercially and not commonly misidentified.
Mycoplasma contamination	Cell lines are tested monthly for mycoplasma. Results are negative.
Commonly misidentified lines (See ICLAC register)	None.

Plants

Seed stocks	n/a
Novel plant genotypes	n/a
Authentication	n/a
